# A Pilot Study of Whole-Blood Transcriptomic Analysis to Identify Genes Associated with Repetitive Low-Level Blast Exposure in Career Breachers

**DOI:** 10.3390/biomedicines10030690

**Published:** 2022-03-17

**Authors:** Rany Vorn, Katie A. Edwards, James Hentig, Sijung Yun, Hyung-Suk Kim, Chen Lai, Christina Devoto, Angela M. Yarnell, Elena Polejaeva, Kristine C. Dell, Matthew L. LoPresti, Peter Walker, Walter Carr, James R. Stone, Stephen T. Ahlers, Jessica M. Gill

**Affiliations:** 1 School of Nursing and Medicine, Johns Hopkins University, Baltimore, MD 21205, USA; 2 National Institute of Nursing Research, National Institutes of Health, Bethesda, MD 20892, USA; katie.edwards@nih.gov (K.A.E.); kimhy@mail.nih.gov (H.-S.K.); christina.devoto@nih.gov (C.D.); 3 Warrior Recovery Center, Evans Army Community Hospital, Fort Carson, CO 80913, USA; james.t.hentig.CTR@mail.mil; 4 Traumatic Brain Injury Center of Excellence, Fort Carson, CO 80913, USA; 5 General Dynamics Information Technology, Falls Church, VA 22042, USA; 6 Predictiv Care, Inc., Mountain View, CA 94086, USA; sijungyun@yottabiomed.com; 7 Center for Neuroscience and Regenerative Medicine, Uniformed Services University of the Health Sciences, Bethesda, MD 20814, USA; chen.lai.ctr@usuhs.edu; 8 Henry M. Jackson Foundation for the Advancement of Military Medicine, Bethesda, MD 20817, USA; 9 Military Emergency Medicine Department, Uniformed Services University of the Health Sciences, Bethesda, MD 20814, USA; angela.yarnell@usuhs.edu; 10 VA San Diego Healthcare System, San Diego, CA 92161, USA; polejaeva@ufl.edu; 11 Department of Psychology, Pennsylvania State University, State College, PA 16802, USA; kzd64@psu.edu; 12 Center for Military Psychiatry and Neuroscience, Walter Reed Army Institute of Research, Silver Spring, MD 20910, USA; matthew.l.lopresti.mil@army.mil (M.L.L.); walter.s.carr.civ@mail.mil (W.C.); 13 Program Executive Office Unmanned and Small Combatants, NAVSEA, Washington, DC 20003, USA; peter.b.walker.mil@us.mail.mil; 14 Oak Ridge Institute for Science and Education, Oak Ridge, TN 37830, USA; 15 Department of Radiology and Medical Imaging, University of Virginia, Charlottesville, VA 22903, USA; jrs7r@virginia.edu; 16 Operational and Undersea Medicine Directorate, Naval Medical Research Center, Silver Spring, MD 20910, USA; stephen.t.ahlers.civ@mail.mil

**Keywords:** repetitive low-level blast, experienced breacher, traumatic brain injury

## Abstract

Repetitive low-level blast exposure is one of the major occupational health concerns among US military service members and law enforcement. This study seeks to identify gene expression using microRNA and RNA sequencing in whole-blood samples from experienced breachers and unexposed controls. We performed experimental RNA sequencing using Illumina’s HiSeq 2500 Sequencing System, and microRNA analysis using NanoString Technology nCounter miRNA expression panel in whole-blood total RNA samples from 15 experienced breachers and 14 age-, sex-, and race-matched unexposed controls. We identified 10 significantly dysregulated genes between experienced breachers and unexposed controls, with FDR corrected <0.05: One upregulated gene, *LINC00996* (*long intergenic non-protein coding RNA 996*); and nine downregulated genes, *IGLV3-16* (*immunoglobulin lambda variable 3-16*), *CD200* (*CD200 molecule*), *LILRB5* (*leukocyte immunoglobulin-like receptor B5*), *ZNF667-AS1* (*ZNF667 antisense RNA 1*), *LMOD1* (*leiomodin 1*), *CNTNAP2* (*contactin-associated protein 2*), *EVPL* (*envoplakin*), *DPF3* (*double PHD fingers 3*), and *IGHV4-34* (*immunoglobulin heavy variable 4-34*). The dysregulated gene expressions reported here have been associated with chronic inflammation and immune response, suggesting that these pathways may relate to the risk of lasting neurological symptoms following high exposures to blast over a career.

## 1. Introduction

Blast exposure is a prominent feature of the Iraq and Afghanistan conflicts due to the use of improvised explosive devices [1]. The prevalence of blast-exposure injury dramatically increased from 60% (2008) to 74% (2009) in the US military, which accounts for most combat-related casualties [2]. Repetitive high-blast exposure has been associated with neuronal changes as well as cognitive and affective symptoms [3,4]. In addition to these concerns from high-pressure blast exposure, repetitive low-level blast exposure is a major occupational health concern for military and law-enforcement training. Experienced breachers in military and law-enforcement training encounter more than 100 occurrences of repetitive low-level blast overpressure throughout their careers [5]. However, the underlying biological mechanism of repetitive low-level blast exposure and related neurological effects are not well-understood. The lack of understanding of repetitive low-level blast exposure on neurological functioning makes its identification and potential health intervention challenging in a military setting. Previously repetitive low levels of blast exposure have been associated with neurocognitive and neurosensory decline [6,7,8], which positively correlated with blood-based levels of tau, amyloid β (Aβ)40, and Aβ42 proteins [9,10], suggesting that there may be biological changes in the blood that result from these high levels of blast exposure over a career.

To date, a growing number of investigations have been conducted to identify biomarkers following repetitive blast exposure [5,9,10,11,12,13]. Previously we reported the effects of acute blast exposure during military training, which include acute changes in amyloid precursor protein [12], inflammatory markers [interleukin (IL)-6 and tumor necrosis factor-alpha (TNF-α)] [13], and longitudinal changes in DNA methylation [14]. It is important to understand the biological mechanism following repetitive low-level blast exposure to develop interventions in preventing short- or long-term associated symptoms that influence the health-related quality of life of US military service members. To address this critical issue, we performed transcriptome-wide analysis in whole-blood RNA sequencing to identify potential gene-expression activity across the genome in an experienced breacher population with a high number of repetitive low-level blast exposures and an unexposed control group. In addition, we used multiplexed miRNA assays to quantify the levels of microRNA expression in the whole blood of the experienced breacher population and unexposed control group.

## 2. Materials and Methods

### 2.1. Study Protocol

This study was reviewed and approved by the Naval Medical Research Center (NMRC) and the National Institutes of Health (NIH) Institutional Review Boards. All the study procedures were performed at NIH Clinical Center after obtaining written informed consent. The detailed procedure of this study protocol has been published elsewhere [5,15].

### 2.2. Demographic, Clinical History, and Psychometric Testing

All participants were administered demographic and clinical information. Psychometric tests were conducted to assess cognitive domains and symptomology of the participants. The groups are well-matched on age, sex, race, and ethnicity. The Immediate Post-Concussion Assessment and Cognitive Test (ImPACT 2.0) was utilized to evaluate verbal-memory composite, visual-memory composite, reaction-time composite, impulse-control composite, and total symptom composite score [16]. The Brief Symptom Inventory (BSI)-18 is an 18-item scale of psychological distress classified into somatization, depression, anxiety, and global severity index subscales [17]. The Combat Exposure Checklist (CEC) is a self-report scale used to measure the frequency of stressful events experienced during deployments. The Neurobehavioral Symptom Inventory (NSI) was used to assess post concussive symptoms. The NSI is a 22-item self-report scale and has shown both excellent internal consistency (α = 0.95) as well as the ability to differentiate veterans with TBIs from those without [18]. Post-Traumatic Stress Disorder (PTSD) Checklist-Military (PCL-M) was used to assess PTSD symptoms. The PCL-M is a 17-item self-report PTSD-symptom scale with scores ranging from 17 to 85. It has been shown to have high test–retest reliability (r = 0.96) and internal consistency (α = 0.96) in Vietnam veterans [19].

### 2.3. RNA Sequencing and Bioinformatic Analysis

Peripheral blood samples were collected in PAXgene tubes and stored at −80 °C until analyzed. Samples from 29 participants were analyzed using RNA-seq with Illumina’s HiSeq 2500 system, using paired-end sequencing. Each sample has at least 30 million reads—15 million reads for read 1 and 15 million reads for read 2. Each read has 101 bp for its read length. For bioinformatics analysis, we first performed bioinformatics quality control (QC) using FastQC, version 0.11.9. Then, we trimmed 15 bp from 5′-end, and 10 bp from 3′-end, to remove adapter contamination as well as low-quality base calls in 3′-end. We aligned to GRCh38 reference genome using STAR, version 2.7.6a. We counted number of reads mapped to genes using htseq, version 0.11.4. Finally, we found differentially expressed genes using DESeq2, version 1.30.1 with the cutoff of 0.05 on false discovery rate (FDR) adjusted by independent hypothesis weighting. R, version 4.0.3 (10 October 2020) and Bioconductor, version 3.11 was used for analysis.

### 2.4. MicroRNA Profiling and Bioinformatic Analysis

Analysis was performed with nCounter^®^ Human v3 miRNA Expression Panels (NanoString Technologies, Seattle, WA, USA). The expression panel contained 798 miRNA probes; this was the maximum number of probes available for analysis in human samples. The probes were incorporated in the code sets and used for analysis along with positive and negative controls. All hybridizations took place at 18 h ± 30 min, and all counts were obtained from nCounter^®^ Digital Analyzer. Raw miRNA data were subtracted from the geometric means of the negative control incorporated in the code sets, and top-100 normalization was performed using the nSolver analysis software (version 4.0, NanoString technologies). Normalized data were analyzed by ROSALIND^®^ (with a HyperScale architecture developed by ROSALIND, Inc. (San Diego, CA, USA). Read-distribution violin plots, identity heatmaps, and sample MDS plots were generated as part of the QC step. The limma R library [20] was used to calculate fold changes and *p*-values and perform optional covariate correction.

### 2.5. Statistical Analysis

Statistical analysis was conducted with SPSS version 28.0 (IBM Corp., Armonk, NY, USA). Demographic and clinical characteristics were compared between the experienced breacher and control groups using chi-square and independent-samples *t*-test. Pearson correlation coefficient was performed to assess the association of the interested study variables. Statistical tests were two-tailed and *p* < 0.05 was considered a significant difference.

## 3. Results

### 3.1. Demographic and Clinical Characteristic

The participants recruited for this study were well-matched on demographic characteristics between the unexposed control (N = 14) and experienced breacher (N = 15) groups (Table 1). The majority of participants were white, military personnel, and had a mean age of 40 years. There were no differences in demographics including age, sex, and ethnicity between experienced breachers and unexposed controls. The mean values of self-reported career breachers were 4659.20 breaching blast exposures in the experienced breacher group and 5.86 breaching blast exposures in the unexposed control group over their careers.

Self-reports of several symptoms were different between the experienced breacher and unexposed control groups (Table 2). A total of 10 out of 15 experienced breachers reported having memory problems and ringing in the ears, whereas only 4 out of 14 reported these symptoms in the unexposed control group (*p* = 0.04). A total of 8 out of 15 experienced breachers reported having irritability problems, and only 2 out of 14 unexposed controls reported irritability (*p* = 0.027). A total of 9 out of 15 experienced breachers reported having concentration problems, whereas only 2 were reported in unexposed controls (*p* = 0.011). Although it did not reach significance, sleep problems were reported in 9 experienced breachers as compared to 5 unexposed controls. Self-reports of headaches and depression were not different between experienced breachers and unexposed controls.

PCL-M scores were higher in experienced breachers when compared with unexposed controls (*p* = 0.029), indicating increased PTSD-related symptoms, although these levels do not meet clinical criteria for PTSD diagnosis (>44 PTSD cutoff) (Table 2). There was no difference in BSI subscale score, including somatization, depression, anxiety, and global severity index scores in experienced breachers when compared with unexposed controls. There was a significant difference between the groups in visual memory (*p* = 0.009) and reaction time (*p* = 0.034).

The results of Pearson correlation coefficients between behavioral symptoms and number of blast exposures are shown in Table 3. The number of blast exposures was positively correlated with BSI subscale, including somatization (*ρ* = 0.399, *p* = 0.032), depression (*ρ* = 0.430, *p* = 0.020), anxiety (*ρ* = 0.496, *p* = 0.006), and global severity index (*ρ* = 0.413, *p* = 0.026).

### 3.2. Differential microRNA Expression Differences between Experienced Breacher vs. Unexposed Control

**Figure 1:**
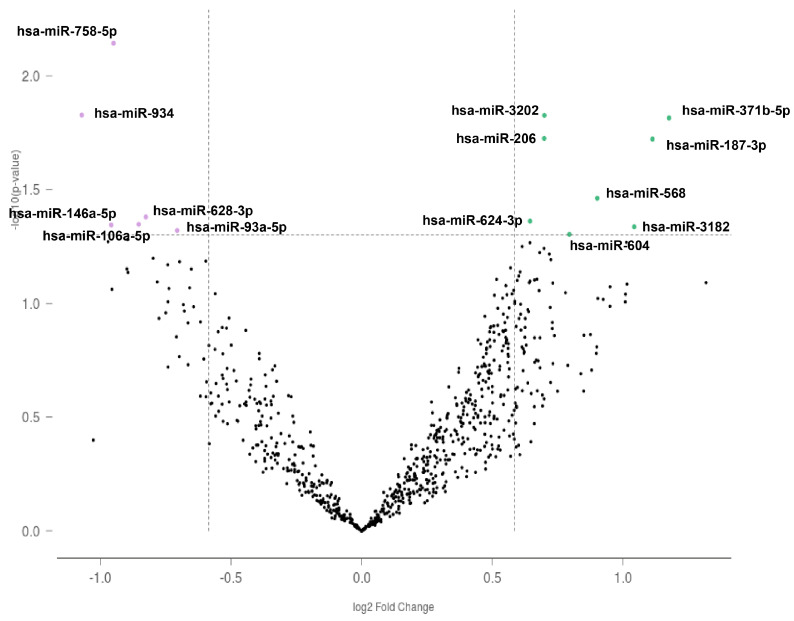
Volcano plot for differentially expressed microRNAs between experienced breacher vs. unexposed control. Green dots indicate genes that are upregulated, and purple dots indicate genes that are downregulated.

We identified 14 miRNAs differentially expressed in experienced breachers compared to unexposed controls (*p* < 0.05). Among them, eight miRNAs were upregulated and six miRNAs were downregulated in the experienced breachers compared with the unexposed controls. These microRNAs were not significantly different after FDR correction. The volcano plot of differentially expressed miRNAs is shown in Figure 1 and the fold change of each probe is presented in Table 4.

### 3.3. Differential Gene Expression between Experienced Breacher vs. Unexposed Control

We performed whole-blood RNA-seq in experienced breacher and unexposed control individuals. The comparison between experienced breacher vs. control shows one upregulated gene and nine downregulated genes. We identified one upregulated gene, *long intergenic non-protein coding RNA 996 (LINC00996*), and 9 downregulated genes, namely *immunoglobulin lambda variable 3-16 (IGLV3-16)*, *CD200 molecule (CD200)*, *Leukocyte immunoglobulin-like receptor B5 (LILRB5)*, *ZNF667 antisense RNA 1 (ZNF667-AS1)*, *leiomodin 1 (LMOD1)*, *contactin-associated protein 2 (CNTNAP2)*, *envoplakin (EVPL)*, *double PHD fingers 3 (DPF3)*, and *immunoglobulin heavy variable 4-34 (IGHV4-34)* in the experienced breacher group compared to control with the multiple corrected threshold of FDR < 0.05. Differential gene expression with fold changes and adjusted *p*-values are shown in Table 5.

## 4. Discussion

In this study, we report significant transcriptome differences in whole-blood associated with repetitive low-level blast exposures compared to unexposed controls. Differentially expressed genes reported are related to inflammation and immune-response process. In addition, the number of blast exposures are strongly correlated with clinical symptoms of BSI-somatization, BSI-anxiety, BSI-depression, and BSI-global severity scores. Dysregulation of these genes may associate with persistent clinical symptoms following repetitive blast exposures. These findings provide some initial insights into the biological changes related to repetitive low-level blast exposure.

Our finding of downregulated *CD200* may play a role in chronic inflammation within the CNS by releasing inflammatory cytokines after exposure to blast. *CD200* is an immune inhibitory molecule which is highly expressed in neurons and plays a critical role in inhibiting microglia activation [21]. The downregulation of *CD200* expression has been observed in both chronic active and inactive multiple sclerosis lesions from postmortem brains in patients [22]. Downregulation of *CD200* in our study may reflect the chronic neuroinflammation activity by microglia activation in individuals exposed to repetitive low levels of blast. Activated microglia is a neuroinflammatory process that affects the astrocytes, leading to astrogliosis, which was observed in the elevation of glial fibrillary acidic protein reported in the preclinical model of repetitive low-level blast exposure [11]. The ongoing inflammatory activity in the CNS can be detected in the peripheral circulation. In support of this, we also report the downregulation of *IGLV3-16*, *IGHV4-34*, and *LILRB5* genes, which are linked to immune-system response and may play an important role in proinflammatory cytokine production [23,24]. Previously, we observed the elevation of plasma IL-6 and TNF-α proteins in a military training population with >5 psi blast exposure compared with low-level <2 psi blast exposure [13]. More recently, analysis of inflammatory proteins in this population of experienced breachers and control subjects showed increases in brain-derived extracellular vesicles (EVs) for IL-6 and TNF-a with a corresponding decrease in IL-10 EVs (unpublished data). These findings further support the important role of *CD200* in the peripheral and CNS activity in response to inflammation after exposure to a high number of blasts during a career.

In addition, we reported a downregulation of *CNTNAP2* gene expression in this cohort. *CNTNAP2* encodes CASPR2, a transmembrane protein associated with voltage potassium channels and a neurexin superfamily protein that plays a critical role in neurodevelopment [25,26]. The *CNTNAP2* gene, located on chromosome 7q35, is one of the largest genes in the human genome [27,28]. Multiple mutations within the gene have been identified and are characterized with a set of neurologically related phenotypes that include intellectual disability, seizures, and language impairment. Additionally, mutations have been clinically associated with neurological disorders such as autism spectrum disorder and Pitt Hopkins-like Syndrome 1 [29,30,31]. Most identified mutations within *CNTNAP2* were heterozygous, indicating that a single allele disruption could be sufficient to cause disorder and deficit. Preclinical models examining homozygous disruption and complete loss of function in the *CNTNAP2* gene have demonstrated an exacerbated and severe neurodevelopmental and neurocognitive deficit outcome [32].

Currently, the majority of investigations revolve around *CNTNAP2* gene mutations with large deletions, which likely lead to a nonfunctional protein product. However, our results demonstrate that in our cohort of experienced breachers, who have repeated low-level blast exposure, *CNTNAP2* gene expression is downregulated. Interestingly, studies have identified individuals with deletions predicted to not interfere with the protein product, or with a deletion to an upstream promoter [33,34,35]. Unlike the majority of deletions that result in a loss of function, these mutations resulted in decreased expression [36], reduced protein function, and displayed more moderate phenotypes including epilepsy, schizophrenia, obsessive compulsive disorder, Tourette syndrome, and attention deficit hyperactivity disorder [33,35,37,38]. Furthermore, downregulation of *CNTNAP2* has been clinically implicated in neurodegeneration, and found significantly downregulated in a cohort of Alzheimer’s patients [39]. Thus, our findings suggest that dysregulation of this gene may be implicated in neurocognitive declines in repetitive low-level blast exposure. Longitudinal follow-up and further analysis of our cohort’s psychiatric health could potentially elucidate an association of blast-related *CNTNAP2* downregulation with other psychological disorders. Additionally, future investigations should examine if differential *CNTNAP2* expression is acutely impacted by blast exposures and possible roles in symptom development in a more lasting manner.

In summary, this preliminary study suggests that occupational exposure to repetitive low-level blasts is associated with dysregulation of gene expression in whole-blood samples. The differentially expressed genes are largely associated with the chronic inflammation process, and linked to various neurological disorders. A major strength of this study is that it is the first to analyze differential gene expression in the whole blood of a unique population with similar occupational factors between experienced breacher and unexposed control groups. Although this is the first study to provide molecular insight into repetitive low level blast exposure, it was constrained by a small sample size and only one timepoint. In addition, the experienced breachers group had significantly higher PCL-M scores compared to unexposed controls, indicating increased PTSD-related symptoms. Higher scores of PTSD-related symptoms may have a significant role on these genes’ expression levels, although these levels do not meet clinical criteria for PTSD diagnosis. Our findings suggest that need for future studies to be undertaken in larger cohorts over time.

Despite these limitations, this is the first study to the best of our knowledge to quantify microRNAs and mRNAs, and this provides initial insights into the pathophysiological mechanism of repetitive low-level blast exposure. Replicating these findings in a larger cohort may provide potential biomarkers and therapeutic targets for experienced breacher populations.

## Figures and Tables

**Table 1:** Demographic characteristics of study participants.

	Unexposed Control (N = 14)	Experienced Breacher (N = 15)	Significance
Age, mean (SD)	38.86 (7.81)	41.60 (8.42)	*t* = 0.907, *p* = 0.372
Sex (Male), N (%)	14 (100)	15 (100)	N/A
Race, N (%)			
White	12 (85.7)	13 (86.7)	χ^2^ = 2.008, *p* = 0.571
Black	1 (7.1)	0 (0.0)	
Asian	1 (7.1)	1 (6.7)	
American Indian/Alaskan	0 (0)	1 (6.7)	
Ethnicity (Non-Hispanic), N (%)	13 (92.9)	15 (100)	χ^2^ = 1.110, *p* = 0.483
Type of Service, N (%)			
Military	10 (71.4)	10 (66.7)	N/A
Civilian Law Enforcement	4 (28.6)	5 (33.3)	
Duration of service, mean (SD)	13.71 (7.12)	18.40 (6.82)	*t* = −1.809, *p* = 0.082
Total blast exposures, mean (SD)	5.86 (10.42)	5659.20 (9649.52)	***t* = −2.269, *p* = 0.040**
Breaches in career, N (%)			
0	13 (92.9)	0 (0.0)	N/A
10–39	1 (7.1)	0 (0.0)	
100–199	0 (0.0)	1 (6.7)	
200–399	0 (0.0)	1 (6.7)	
400+	0 (0.0)	13 (86.7)	
Breaches in past year, N (%)			
0	14 (100)	2 (13.3)	N/A
1–9	0 (0.0)	2 (13.3)	
10–39	0 (0.0)	1 (6.7)	
40–99	0 (0.0)	1 (6.7)	
100–199	0 (0.0)	3 (20.0)	
200–399	0 (0.0)	3 (20.0)	
400+	0 (0.0)	3 (20.0)	

N/A: Not Applicable.

**Table 2:** Clinical symptoms of study participants.

	Unexposed Control (N = 14)	Experienced Breacher (N = 15)	Significance
Headaches, Yes, N (%)	2 (14.3)	2 (13.3)	χ^2^ = 0.006, *p* = 0.941
Memory problem, Yes, N (%)	4 (28.6)	10 (66.7)	**χ^2^ = 4.209, *p* = 0.040**
Ringing in ears, Yes, N (%)	4 (28.6)	10 (66.7)	**χ^2^ = 4.209, *p* = 0.040**
Sleep problems, Yes, N (%)	5 (35.7)	9 (60.0)	χ^2^ = 1.710, *p* = 0.191
Irritability, Yes, N (%)	2 (14.3)	8 (53.3)	**χ^2^ = 4.887, *p* = 0.027**
Depression, Yes, N (%)	3 (21.4)	3 (20.0)	χ^2^ = 0.009, *p* = 0.924
Concentration problems, Yes, N (%)	2 (14.3)	9 (60.0)	**χ^2^ = 6.428, *p* = 0.011**
PCL-M, mean (SD)	20.64 (4.48)	26.07 (7.69)	***t* = −2.338, *p* = 0.029**
NSI, mean (SD)	16.86 (5.29)	16.80 (6.70)	*t* = −0.025, *p* = 0.980
BSI subscale, mean (SD)			
Somatization	45.29 (4.41)	49.33 (7.46)	*t* = −1.762, *p* = 0.089
Depression	45.21 (6.40)	46.00 (7.05)	*t* = −0.313, *p* = 0.756
Anxiety	44.07 (5.81)	44.60 (8.95)	*t* = −0.187, *p* = 0.853
Global severity index	43.50 (6.40)	46.40 (9.15)	*t* = −0.986, *p* = 0.333
ImPACT, mean (SD)			
Verbal memory	92.21 (6.33)	92.13 (6.53)	*t* = 0.034, *p* = 0.973
Visual memory	68.00 (9.12)	78.60 (11.06)	***t* = −2.804, *p* = 0.009**
Visual motor speed	27.83 (3.25)	27.33 (4.57)	*t* = 0.337, *p* = 0.738
Reaction time	0.57 (0.07)	0.64 (0.10)	***t* = −2.213, *p* = 0.034**
Impulse control	0.07 (0.28)	0.33 (0.62)	*t* = −1.500, *p* = 0.150
Total symptom	4.71 (6.75)	12.40 (17.59)	*t* = −1.573, *p* = 0.133

PCL-M, Post-Traumatic Stress Disorder Checklist-Military; NSI, Neurobehavioral Symptom Inventory; BSI, Brief Symptom Inventory.

**Table 3:** Pearson Correlation Coefficients of Study Variables.

	Number of Blast Exposures		CEC Total Score	
	*ρ*	*p*	*ρ*	*p*
BSI-somatization	**0.399**	**0.032**	**0.377**	**0.044**
BSI-depression	**0.430**	**0.020**	−0.003	0.990
BSI-anxiety	**0.496**	**0.006**	−0.056	0.773
BSI-global severity index	**0.413**	**0.026**	0.162	0.402
ImPACT-verbal memory	−0.055	0.776	−0.114	0.557
ImPACT-visual memory	0.053	0.784	**0.508**	**0.005**
ImPACT-visual motor speed	−0.324	0.087	0.133	0.491
ImPACT-reaction time	**0.448**	**0.015**	0.264	0.166
ImPACT-impulse control	0.202	0.293	−0.069	0.722
ImPACT-total symptom	0.243	0.204	0.223	0.244

BSI, Brief Symptom Inventory; CEC, Combat Exposure Checklist; ImPACT, Immediate Post-Concussion Assessment and Cognitive Test.

**Table 4:** MicroRNA differential expression between experienced breacher vs unexposed control.

Probe Name	Log2FC	*p*-Value	FDR
hsa-miR-371b-5p	1.177	0.015	0.848
hsa-miR-187-3p	1.113	0.019	0.848
hsa-miR-3182	1.043	0.046	0.848
hsa-miR-568	0.901	0.035	0.848
hsa-miR-604	0.795	0.049	0.848
hsa-miR-3202	0.699	0.015	0.848
hsa-miR-206	0.699	0.019	0.848
hsa-miR-624-3p	0.644	0.043	0.848
hsa-miR-93-5p	−0.706	0.048	0.848
hsa-miR-628-3p	−0.826	0.042	0.848
hsa-miR-106a-5p	−0.853	0.045	0.848
hsa-miR-758-5p	−0.950	0.007	0.848
hsa-miR-146a-5p	−0.959	0.045	0.848
hsa-miR-934	−1.071	0.015	0.848

**Table 5:** Gene-expression differences between experienced breacher vs unexposed control.

Gene Symbol	Gene Name	Log2FC	FDR
*IGLV3-16*	*Immunoglobulin lambda variable 3-16*	−1.880	0.002
*CD200*	*CD200 molecule*	−0.879	0.024
*LILRB5*	*Leukocyte immunoglobulin-like receptor B5*	−1.689	0.024
*ZNF667-AS1*	*ZNF667 antisense RNA 1*	−0.974	0.024
*LMOD1*	*Leiomodin 1*	−2.688	0.025
*CNTNAP2*	*Contactin-associated protein 2*	−1.715	0.030
*EVPL*	*Envoplakin*	−2.263	0.030
*DPF3*	*Double PHD fingers 3*	−1.542	0.039
*LINC00996*	*Long intergenic non-protein coding RNA 996*	0.866	0.039
*IGHV4-34*	*Immunoglobulin heavy variable 4-34*	−1.250	0.043

## Data Availability

The datasets presented in this study can be found in online repositories at Gene Expression Omnibus with the access number GSE193952. The anonymized clinical data that support the findings of this study are available upon reasonable request from any qualified investigator to the corresponding author.
